# Inverted Toe Nails

**Published:** 1876-05

**Authors:** 


### INVERTED TOE-NAILS.

A PERSON lately died, in Boston, from the effects of the toe-nail "growing into the flesh," as it is described, at the edges.

It is not the growing of the nail into the flesh; but it is the flesh, at that point, becoming irritated and swollen which racks the system with pain, and makes walking impossible, if relief is not obtained. Some medical journals still advise the horrible practice of dragging the nail out of its socket with pincers; but we have known this expedient to fail. The difficulty is occasioned by wearing shoes too narrow for the foot across the toes.

Many an excellent receipt becomes worthless in the hands of those who try it, in consequence of not making the experiment fairly. We were once called to see the toe of a young mother, who had been told that a bit of soft cotton, put under the toe-nail, would cure it;, and it certainly will, if applied properly: but, in her case, it proved a great aggravation. She had been suffering a whole year; and, at the time, her foot was in a terrible condition. She had crowded a wad of cotton between the part of the toe-nail which grows beyond the skin, *in the centre of the arch;* that elevated the centre of the arch, and depressed the ends of it, thus increasing the torture.

Do not cut off the corners of the nail; let them grow out with the other projecting portion, so as to receive a soft string of raw cotton, made by twisting it with the thumb and finger. Put this under the breadth of the nail which extends beyond the toe, so that, at the edges or corner, the soft cotton-string may be between the nail and the skin: this string should be long enough at both ends to be drawn backwards towards the body, and wound around the toe in the rear of the nail; and should be done in such a manner, that it shall be kept in its place. A new and thicker string should be applied every morning.

In addition, the centre of the nail, along its whole length, should be notched out, or scraped with a piece of broken glass—scraped down to the very quick, in as narrow a line as possible—the object being, to make the nail at the centre as thin as possible, making it yielding. Thus, while the cotton raises the edges from the flesh, the centre yields, because the arch is broken. But the greatest benefit is done by the cotton in another direction; and we enter into the philosophical explanation of it, that it may be the better remembered, and intelligently applied. If a strap is tightened around the human arm, more and more, daily, in a few days there will be scarcely any thing under the strap, except the "skin and bone." This is owing to a physiological law, that pressure produces absorption; that is, an imperceptible removal of the soft parts pressed against. In the case in hand, the roll of cotton presses against the toe-nail above, and the swollen, "proud," angry flesh beneath; and this pressure not only causes a removal of particles already there, but largely prevents the blood from entering and increasing the inflammation. A judicious use of the cotton-yarn would alone be sufficient; but the scraping of the nail at the crown of the arch, lengthwise the nail, expedites the cure; and if, besides this, a person will have the moral courage to eat but half the usual amount, daily; or, to make the rule more explicit, will live literally on bread and water, until the trouble is mainly removed—the perfect cure will be completed in about half the time, and with half the discomfort.

If there is much heat or hardness of the parts, ease will be more speedily obtained, if they are kept moist all the time, for the first few days. To this end, a warm poultice may be applied every hour, from daylight until bed-time; and a thicker one during the night, to keep moist longer. We know of no better, safer, simpler, more accessible poultice, for this and other purposes, than one made of stale, light bread, and sweet milk. Its superiority lies in its being always at hand, and in its keeping moist longer, and being more readily re-moistened, than others.

